# Resveratrol Inhibits Hepatic Stellate Cell Activation via the Hippo Pathway

**DOI:** 10.1155/2021/3399357

**Published:** 2021-10-13

**Authors:** Chunxue Li, Rongrong Zhang, Yating Zhan, Jianjian Zheng

**Affiliations:** Key Laboratory of Diagnosis and Treatment of Severe Hepato-Pancreatic Diseases of Zhejiang Province, The First Affiliated Hospital of Wenzhou Medical University, Wenzhou 325000, China

## Abstract

Liver fibrosis, which results from chronic liver injury due to factors such as chronic alcohol consumption, hepatitis virus infections, and immune attacks, is marked by excessive deposition of extracellular matrix (ECM). Resveratrol (Res), a polyphenol phytoalexin, has been demonstrated to show anti-inflammatory, antioxidative, antiproliferative, and chemopreventive activities. In recent years, Res has been found to inhibit liver fibrosis. Enhanced Hippo pathway activation has also been reported to inhibit tumor progression and liver fibrosis. In the present study, the role of the Hippo pathway in mediating the effects of Res on hepatic stellate cells (HSCs) was examined. We found that Res significantly suppresses HSC proliferation, reducing the cell index. Res induced HSC inactivation, reducing collagen deposition and *α*-smooth muscle actin (*α*-SMA) expression. In addition, Res contributed to HSC apoptosis, upregulating Bax and downregulating Bcl-2 expression. Notably, the Hippo pathway was involved in the Res-mediated suppression of HSC activation. Res enhanced the activation of the Hippo pathway and reduced yes-associated protein (YAP) and transcriptional coactivator with the PDZ-binding motif (TAZ) expression. Interestingly, the YAP overexpression inhibited Res-induced HSC inactivation and apoptosis. In conclusion, these results demonstrate that Res inhibits HSC activation, at least in part, via the Hippo pathway. The present study indicates a new antifibrotic mechanism of Res and provides novel insights into Hippo-mediated HSC apoptosis and HSC activation in liver fibrosis.

## 1. Introduction

Liver fibrosis is a result of immoderate tissue repair responses after chronic liver injury and is characterized by excessive accumulation of extracellular matrix (ECM) components, especially type I collagen [1]. Generally, liver fibrosis is caused by viruses, drugs, alcoholism, metabolic disorders, and immune attacks [2]. If left untreated, persistent liver fibrosis can progress to cirrhosis and even hepatic carcinoma [3]. Accumulating evidence has shown that activated hepatic stellate cells (HSCs) secrete the vast majority of ECM components, thereby accelerating the progression of liver fibrosis [4]. Therefore, the activation of HSCs plays a central role in liver fibrosis, and inhibiting HSC activation and inducing HSC apoptosis are considered appropriate strategies for antifibrosis therapy.

Resveratrol (Res, 3,5,4′-trihydroxystilbene) is a polyphenolic compound widely present in nuts and grapes [5]. Res has been demonstrated to show various beneficial effects, including antioxidant, anti-inflammatory, antiproliferative, and lipid-lowering activities [6] [7]. A growing body of evidence has demonstrated that Res plays a protective role against liver disease. For example, Hosseini et al. demonstrated that Res can attenuate nonalcoholic fatty liver disease via the demethylation of the Nrf2 promoter [8]. Zhang et al. found that Res contributes to the prevention of liver fibrosis by modulating NF-*κ*B and the PI3K/Akt pathway [9]. Further, Res has been demonstrated to alleviate liver fibrosis by inducing autophagy [10]. However, the mechanisms underlying the effects of Res on liver fibrosis are still not fully clear.

The Hippo pathway, a highly conserved signaling pathway initially identified in *Drosophila melanogaster*, consists of kinase cascades [11]. In recent years, several studies have indicated that the Hippo pathway is necessary for the regulation of cell cycle progression, proliferation, apoptosis, and differentiation [12]. Abnormal activation of the Hippo pathway is associated with various human diseases, including inflammation, fibrosis, and cancers [13]. Yes-associated protein (YAP) and transcriptional coactivator with PDZ-binding motif (TAZ), the two main effectors of the Hippo pathway, are generally downregulated when the Hippo pathway is activated [14]. In addition, YAP/TAZ is considered oncogenes in the development and progression of cancers [15]. Importantly, an obvious accumulation of nuclear YAP/TAZ has been observed in myofibroblasts and HSCs [16]. Recently, the Hippo pathway has been reported to participate in the liquiritigenin-mediated suppression of liver fibrosis [17]. However, the contribution of the Hippo pathway to the effects of Res on HSC inactivation remains to be elucidated. To this end, in the present study, we examined the role of the Hippo pathway in the effects of Res on HSCs.

## 2. Materials and Methods

### 2.1. Cell Culture and Treatment

A human HSC line (LX-2) was purchased from the Cell Bank of the Chinese Academy of Sciences. LX-2 cells were cultured in RPMI-1640 medium supplemented with 10% fetal bovine serum (Thermo, USA) and 1% penicillin/streptomycin (Corning, USA). LX-2 cells were treated with 60 *μ*M Res (Yuanye Bio-Technology, Shanghai, China) for 24 h. One group of Res-treated cells was also treated with 5 *μ*M XMU-MP-1, an inhibitor of the Hippo pathway kinases MST1/2, for 24 h.

### 2.2. Animals and Treatment

After 7 days of adaptive feeding, 18 male C57BL/6 J mice (6–8 weeks old, 18–22 g) were randomly divided into the control (*n* = 6), carbon tetrachloride (CCl_4_, Sigma-Aldrich) (*n* = 6), and Res treatment groups (*n* = 6). Mice from the CCl_4_ group received intraperitoneal injections of 10% CCl_4_ (dissolved in olive oil) at a dose of 10 *μ*l/g, twice a week for 8 weeks. Mice from the control group received intraperitoneal injections with an equivalent volume of olive oil. Mice from the Res treatment group received twice-weekly injections of CCl_4_ plus oral Res treatment (20 mg/kg dissolved in 2% DMSO and saline) [18]. At the end of the experiment, mice were sacrificed using 10% chloral hydrate injections (0.01 ml/g, i.p.). This study was approved by the Ethics Committee of the First Affiliated Hospital of Wenzhou Medical University.

### 2.3. Cell Proliferation Analysis

Cell proliferation was measured using the CCK-8 Kit (Dojindo, Japan). Briefly, cells were inoculated in 96-well plates at a density of 5,000 cells per well. After treatment with different concentrations of Res for 24 h, the cells were incubated with 10 *μ*l of CCK-8 reagent per well at 37°C for 3 h. Subsequently, the absorbance was measured at 450 nm to determine the cell proliferation rate.

### 2.4. Real-Time Cell Proliferation Assay (RTCA)

Cell proliferation was analyzed using RTCA [19]. First, 50 *μ*l of cell culture medium was added to E-Plate 16 dishes (ACEA Biosciences Inc, CA, USA), and the background impedance was measured and displayed as the cell index. Then, cells were seeded in these dishes at a density of 5,000 cells per well, and 200 *μ*l of complete medium was added. After 24 h, cells were treated with 60 *μ*M Res. The cell index, representing the change in impedance and the proliferation of cells, was then obtained.

### 2.5. Western Blot Analysis

Total protein was extracted using RIPA lysis buffer (Beyotime, China). The total protein concentration was determined using the BCA protein assay. The proteins (10 *μ*l) were separated using 10% SDS–PAGE and transferred to a 0.2 *μ*m PVDF membrane. Then, the nonspecific binding sites on the membranes were blocked by incubating the membranes with 5% skimmed milk at room temperature for 2 h. Subsequently, the membranes were incubated with primary antibodies at 4°C overnight. The following day, the membranes were incubated with the secondary antibody (Rockland, Limerick, PA, USA) at room temperature for 1 h. GAPDH served as the internal control.

### 2.6. Immunofluorescence Staining

Immunofluorescence staining was performed as previously described [20]. In brief, cells were fixed in 4% paraformaldehyde for 15 min and permeabilized with 0.5% Triton X-100 in PBS for 15 min. After blocking with 5% BSA in PBS for 1 h at 37°C, cells were incubated with the following primary antibodies overnight at 4°C in a humidified chamber: anti-*α*-smooth muscle actin (*α*-SMA) (1 : 500), anti-type I collagen (1 : 100), anti-YAP (1 : 100), and anti-TAZ (1 : 100). On the next day, the cells were incubated with secondary antibodies conjugated with Alexa Fluor 488 (Invitrogen). Nuclei were stained using 4,6-diamidino-2-phenylindole (DAPI), and cells were observed using a microscope (Leica Microsystems GmbH, Wetzlar Germany).

### 2.7. Quantitative Real-Time PCR (qRT-PCR)

Total RNA was extracted using the RNA simple Total RNA Kit (Tiangen Biotech, China). Following the manufacturer's instructions, reverse transcription was performed using a reverse transcription kit (Vazyme Biotech Co., Ltd.). The cDNA was used to measure the gene expression via real-time PCR using the SYBR Green real-time PCR Master Mix (Toyobo, Osaka, Japan). *GAPDH* was used as the internal reference. The primers used were as follows:

YAP sense 5′-AGAACAATGACGACCAATAGCTC-3′, antisense 5′-GCTGCTCATGCTTAGTCCAC-3′; TAZ sense 5′-ACCCACCCACGATGACCCCA-3′, antisense 5′-GCACCCTAACCCCAGGCCAC-3′; alpha-1(I) collagen (Col1A1) sense 5′-TGGCAAAGAAGGCGGCAAAGG-3′, antisense 5′-AGGAGCACCAGCAGGACCATC-3′, *α*-SMA sense 5′-TCGTGCTGGACTCTGGAGATGG-3′, antisense 5′-CCACGCTCAGTCAGGATCTTCATG-3′; and GAPDH sense 5′-AAATCAAGTGGGGCGATGCT-3′, antisense 5′-GTGCTAAGCAGTTGGTGGTG-3′.

### 2.8. Flow Cytometry Analysis

The cell apoptosis rate was analyzed using an Annexin V/7-AAD kit (Biolegend, California, USA). Briefly, cells were treated with Res and collected by centrifugation. Then, they were resuspended in 500 *μ*l binding buffer and mixed with 5 *μ*l APC Annexin V as well as 5 *μ*l 7-AAD staining solution. Finally, the mixture was incubated away from light at room temperature for 5 min, and cells were analyzed using flow cytometry (CytoFlex Beckman CytoFlex, USA).

### 2.9. RNA-Sequencing (RNA-seq)

BioAnalyser (Agilent) was used to determine the RNA concentration, purity, and integrity to ensure the high quality of samples used for transcriptome sequencing. After the samples met the required criteria, the library was constructed, and the main steps were as follows. First, oligo(dT) magnetic beads were used to isolate mRNA from the total RNA. Second, the mRNA was mixed with a fragmentation buffer to obtain smaller fragments (~180 bp) that could serve as templates for cDNA synthesis. First-strand cDNA was synthesized using six-base random primers (random hexamers), and second-strand cDNA was synthesized using dNTPs and RNase H. Subsequently, AMPure XP beads were used to purify the double-stranded cDNA (ds cDNA). Finally, a cDNA sequencing library was created using PCR amplification. After library quality inspection, the Illumina platform was used to perform high-throughput sequencing.

### 2.10. Transfection of *YAP*

For the overexpression of *YAP*, the YAP overexpression plasmid and empty plasmid were purchased from RiboBio (RiboBio, Guangzhou, China). According to the manufacturer's protocol, cells were transfected with the *YAP* overexpression plasmid using the Lipofectamine® 3000 transfection reagent kit (Invitrogen) for 24 h.

### 2.11. Statistical Analysis

All data were expressed as mean ± SD. Comparisons between two groups were performed using Student's *t*-test. Comparisons among multiple groups were performed using one-way analysis of variance. *P* < 0.05 was considered significant. All statistical analyses were performed using SPSS software (version 16.0; SPSS, Chicago, IL).

## 3. Results

### 3.1. Res Attenuates CCl_4_-Induced Liver Fibrosis in Mice

**Figure 1:**
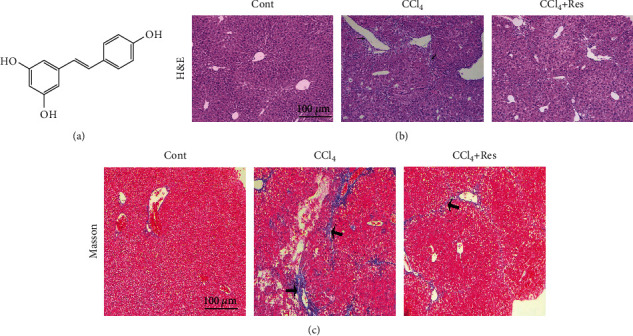
Res attenuates CCl_4_-induced liver fibrosis in mice. (a) Chemical structure of Res. (b) H&E staining. (c) Masson trichome staining. Magnification: ×100. Narrow arrow: inflammatory cell infiltration. Wide arrow: collagen deposition.

The chemical structure of Res is shown in Figure 1(a). To explore whether Res has an antifibrotic effect in vivo, hematoxylin and eosin (H&E) staining and Masson staining assays were performed, and liver fibrogenesis was examined pathologically. As shown in Figure 1(b), H&E staining results showed that liver tissue in the control group had a normal histological structure, the hepatocyte cord was arranged clearly, and there was no infiltration by inflammatory cells. In contrast, the liver tissue in the CCl_4_ group exhibited a mass of inflammatory cell infiltrates and a disordered arrangement of liver cells. Notably, Masson staining showed that treatment with Res could alleviate the collagen deposition caused by CCl_4_ in vivo (Figure 1(c)). These data indicate that Res could inhibit the liver fibrosis induced by CCl_4_ in vivo.

### 3.2. Effect of Res on the Proliferation of HSCs

**Figure 2:**
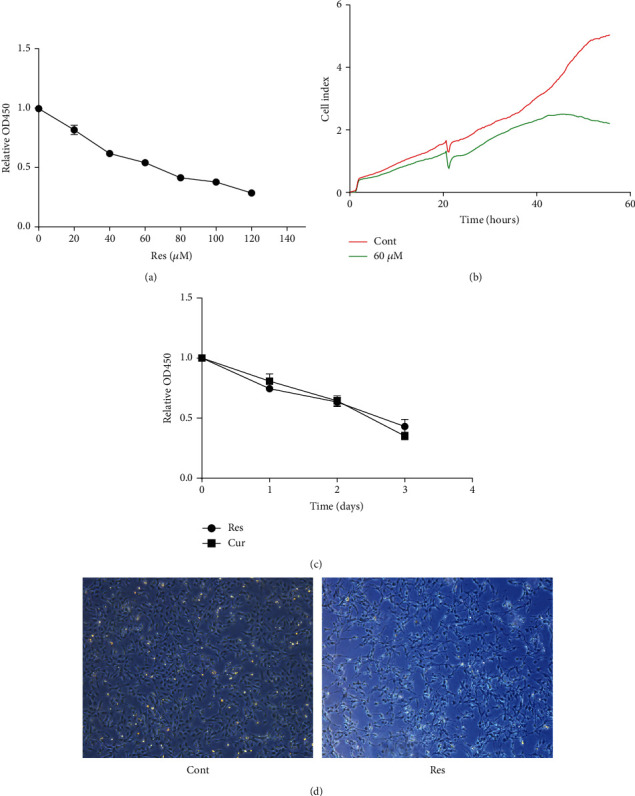
Effect of Res on the proliferation of HSCs. (a) Cell proliferation after treatment with different concentrations of Res determined using the CCK-8 assay. (b) RTCA assay. (c) CCK-8 assay performed to detect cell proliferation in cells treated with Res or Cur. (d) Cell morphology. Magnification: ×25.

Next, the effect of Res on HSC proliferation was evaluated. As shown in Figure 2(a), HSC proliferation was found to be decreased after Res treatment in a dose-dependent manner, and the IC_50_ value of Res was 60 *μ*M. Therefore, 60 *μ*M Res was chosen for the subsequent experiments. In line with this, RTCA indicated that Res reduced the cell index in a time-dependent manner (Figure 2(b)). Recently, curcumin (Cur) has been reported to alleviate liver fibrosis and inhibit HSC activation [21]. Therefore, Cur was used as a positive control in our experiments. Cell proliferation analysis indicated that HSC proliferation was inhibited by Res as well as Cur in a time-dependent manner (Figure 2(c)). As shown in Figure 2(d), Res treatment resulted in a “stellate morphology,” with longer cytoplasmic protrusions. Our data suggest that Res has a suppressive effect on HSC proliferation.

### 3.3. Effects of Res on HSC Transdifferentiation and Collagen Deposition

**Figure 3:**
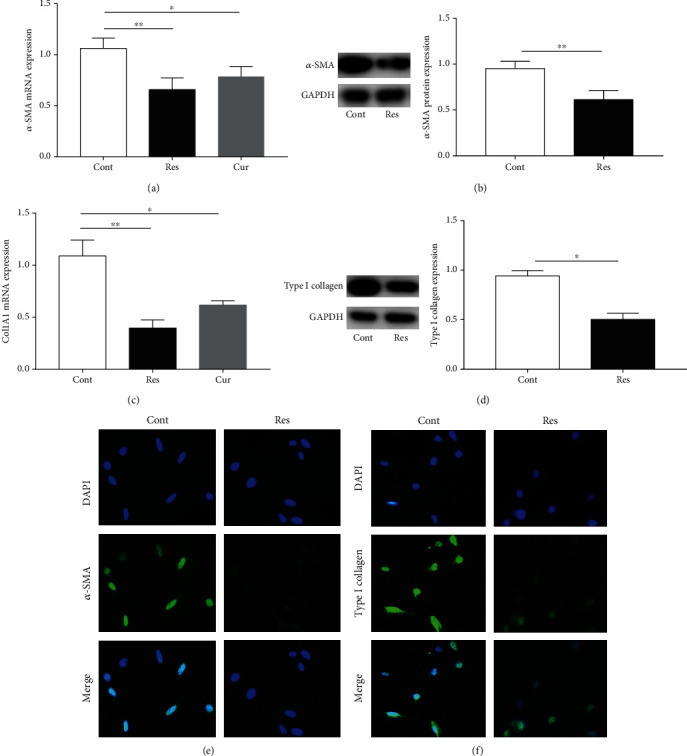
Effects of Res on HSC transdifferentiation and collagen deposition. (a, c) mRNA expression of *α-SMA* and *Col1A1*. (b, d) Protein expression of *α*-SMA and type I collagen. (e, f) Immunofluorescence staining for *α*-SMA and type I collagen. Nuclei are stained blue with DAPI. Original magnification: ×200. Values are expressed as means ± SD. ^∗^*P* < 0.05 and ^∗∗^*P* < 0.01.

Activated HSCs are characterized by increased *α*-SMA expression and enhanced collagen production [22]. Next, we examined the effects of Res on HSC transdifferentiation and collagen expression. Compared with the levels in the control group, there was a significant decrease in *α*-SMA mRNA and protein levels in Res-treated HSCs (Figures 3(a) and 3(b)). Consistent with this, immunofluorescence analysis confirmed the reduction in *α*-SMA levels in Res-treated HSCs, with a decline in green fluorescence intensity (Figure 3(e)). As shown in Figures 3(c) and 3(d), Res treatment caused an obvious reduction in Col1A1 and type I collagen levels. Accordingly, immunofluorescence staining also showed that type I collagen was inhibited by Res (Figure 3(f)). Additionally, we found that Cur suppressed activated HSCs, reducing the expression of *α*-SMA and Col1A1 (Figures 3(a) and 3(c)). Taken together, the data suggest that Res effectively downregulates HSC activation.

### 3.4. Res Induces HSC Apoptosis

**Figure 4:**
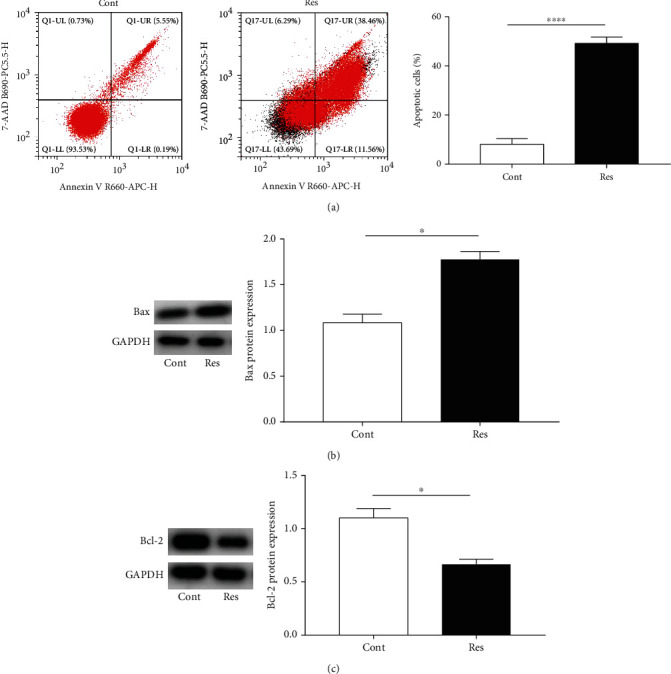
Res induces HSC apoptosis. (a) HSC apoptosis. (b) Bax protein expression. (c) Bcl-2 protein expression. Values are expressed as means ± SD. ^∗^*P* < 0.05 and ^∗∗∗∗^*P* < 0.0001.

The induction of HSC apoptosis is one of the main strategies for antifibrosis treatment. The effect of Res on HSC apoptosis was analyzed using flow cytometry. It is known that Q2 and Q3 indicate late and early cell apoptosis, respectively, and the number of apoptotic cells is equal to the sum of the cells in Q2 and Q3. There was an obvious increase in cell apoptosis in the Res group when compared with the control group (Figure 4(a)). Consistent with the results of flow cytometry analysis, we observed that Res induced the Bax protein expression and decreased Bcl-2 protein expression (Figures 4(b) and 4(c)). These data indicate that Res contributes to HSC apoptosis.

### 3.5. The Hippo Pathway Is Involved in the Effects of Res on HSC Inactivation

**Figure 5:**
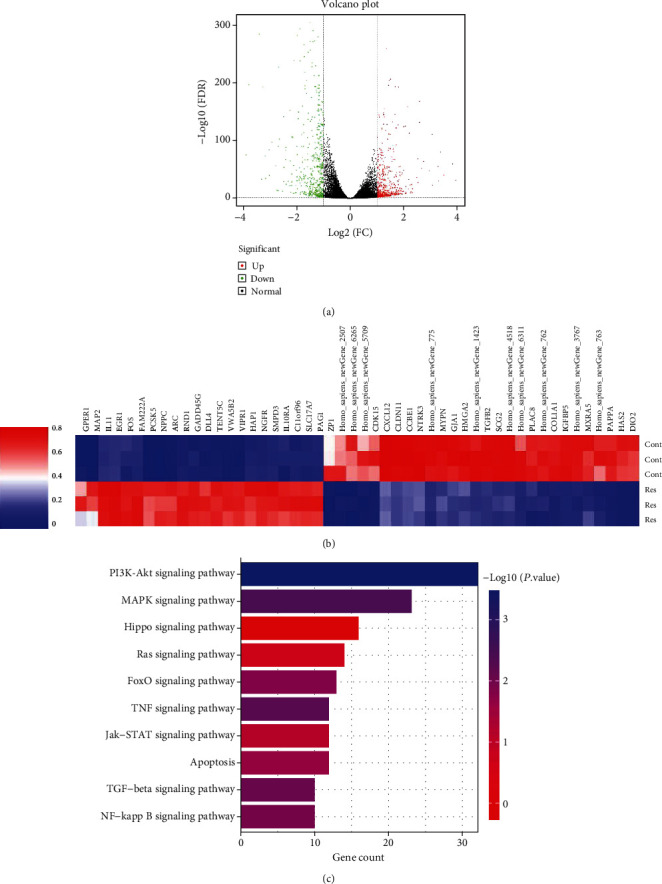
The Hippo pathway is involved in the effects of Res on HSC inactivation. (a) Volcano plot of DEGs; the green dots represent downregulated DEGs, the red dots represent upregulated DEGs, and the black dots represent non-DEGs. (b) Heatmap. (c) KEGG analysis.

In order to determine the molecular mechanism underlying the antifibrotic effects of Res, RNA-seq was performed to identify Res-related pathways. As shown in the volcano map, we identified 479 upregulated genes as well as 458 downregulated genes in Res-treated cells (Figure 5(a)). Next, the top 50 differentially expressed genes (DEGs) were selected and displayed in the heat map (Figure 5(b)). KEGG pathway enrichment analysis showed that three pathways, including PI3K-Akt, MAPK, and Hippo, could mediate the effects of Res on HSC activation. The roles of PI3K-Akt and MAPK pathways have been explored in Res-treated HSCs [9] [23]. In addition, it was found that the Hippo pathway was highly involved in mediating the effects of Res on HSC activation (Figure 5(c)). Therefore, the Hippo pathway was selected for the subsequent experiments.

### 3.6. Res Treatment Enhances the Hippo Pathway

**Figure 6:**
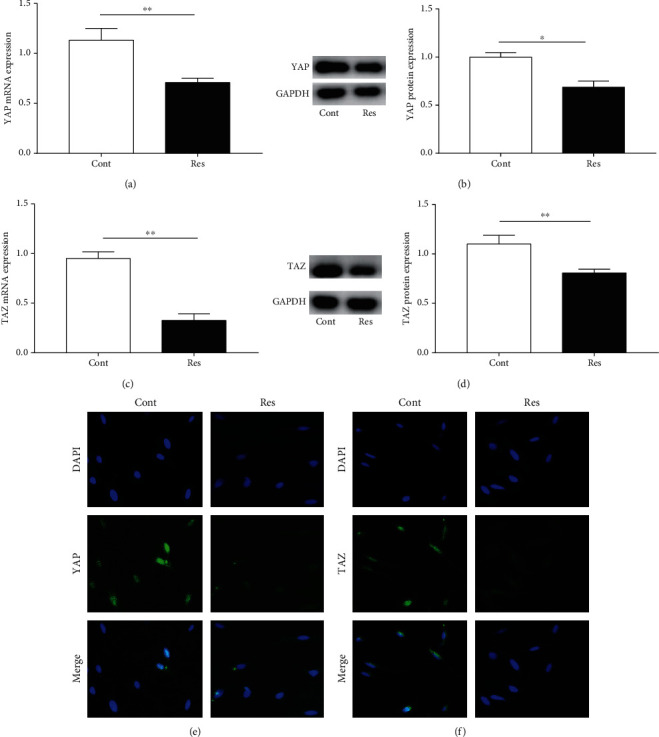
Res treatment enhances the Hippo pathway. (a) *YAP* mRNA expression. (b) YAP protein levels. (c) *TAZ* mRNA expression. (d) TAZ protein levels. (e, f) Immunofluorescence staining for YAP and TAZ. Nuclei are stained blue with DAPI. Original magnification: ×200. Values are expressed as means ± SD. ^∗^*P* < 0.05 and ^∗∗^*P* < 0.01.

The Hippo pathway, generally inactivated during acute tissue damage and a broad range of fibrotic diseases, contributes to tissue repair by promoting cell autonomous proliferation via YAP/TAZ [24]. Next, Hippo pathway-related genes such as *YAP* and *TAZ* were examined in Res-treated cells. As indicated in Figures 6(a) and 6(c), the mRNA levels of *YAP* and *TAZ* were significantly decreased in cells after Res treatment. In line with the mRNA results, the protein expressions of YAP and TAZ were also significantly reduced after Res treatment (Figures 6(b) and 6(d)). Immunofluorescence analysis further confirmed that Res caused a reduction in YAP and TAZ levels, with decreased intensity of green fluorescence (Figures 6(e) and 6(f)). Our results indicate that Res promotes the Hippo pathway.

### 3.7. Loss of Hippo Pathway Activation Attenuates the Res-Induced Inactivation of HSCs

**Figure 7:**
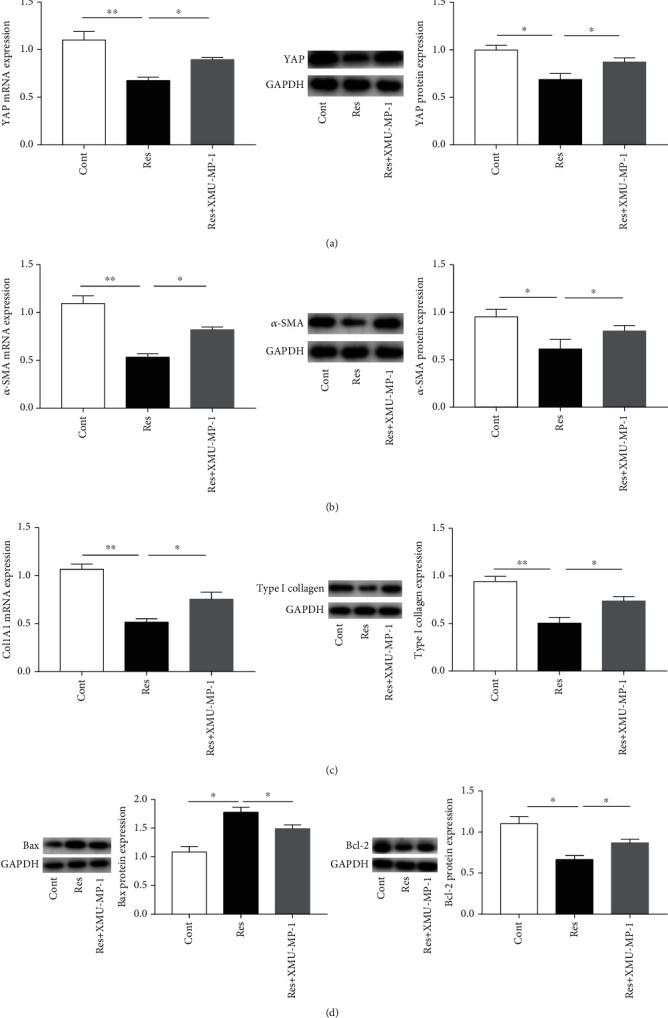
Loss of Hippo pathway activation attenuates the Res-induced inactivation of HSCs. (a) YAP expression. (b) *α*-SMA expression. (c) Col1A1 and type I collagen expression. (d) Bax and Bcl-2 expression. Values are expressed as means ± SD. ^∗^*P* < 0.05 and ^∗∗^*P* < 0.01.

Next, the effects of Hippo pathway inhibition on HSC activation were examined in Res-treated cells. XMU-MP-1, an inhibitor of the Hippo pathway kinases MST1/2, activates the downstream effector molecule YAP and promotes cell growth [25]. XMU-MP-1 treatment induced an increase in the YAP expression in Res-treated cells, suggesting that XMU-MP-1 could inhibit the Hippo pathway (Figure 7(a)). Interestingly, Res-induced HSC inactivation was blocked by XMU-MP-1 treatment (Figures 7(b) and 7(c)). In addition, Res-induced HSC apoptosis was attenuated by the XMU-MP-1-induced inhibition of the Hippo pathway (Figure 7(d)). Taken together, the results demonstrate that reduced Hippo pathway activation inhibits Res-induced HSC inactivation.

### 3.8. YAP Overexpression Inhibits the Effects of Res on the Inactivation of HSCs

**Figure 8:**
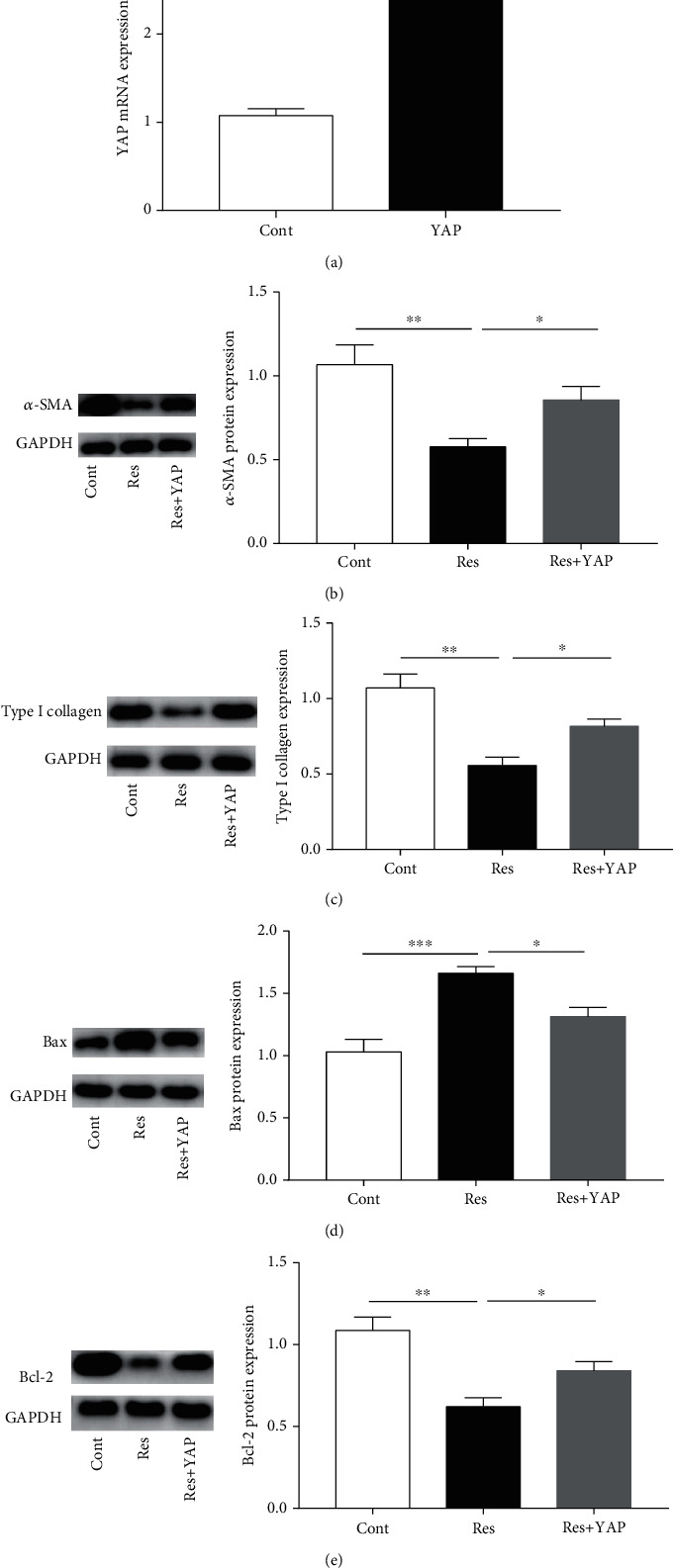
YAP overexpression inhibits the effects of Res on the inactivation of HSCs. (a) *YAP* mRNA expression. (b) *α*-SMA protein expression. (c) Type I collagen expression. (d) Bax protein expression. (e) Bcl-2 protein expression. Values are expressed as means ± SD. ^∗^*P* < 0.05, ^∗∗^*P* < 0.01, and ^∗∗∗^*P* < 0.001.

To further determine whether Res inhibited the activation of HSCs via the Hippo pathway, the *YAP* gene was overexpressed using the *YAP* expression plasmid (Figure 8(a)). In Res-treated cells, the overexpression of YAP contributed to the restoration of *α*-SMA and type I collagen expression, indicating that YAP upregulation leads to enhanced activation of HSCs after Res treatment (Figures 8(b) and 8(c)). Interestingly, compared with the Res group, the YAP overexpression group showed a significant reduction in the expression of the Bax protein (Figure 8(d)). Similarly, the reduction in Bcl-2 protein caused by Res treatment was reversed by the YAP overexpression (Figure 8(e)). All these data demonstrate that Res inhibits the activation of HSCs, at least in part, via the Hippo pathway.

## 4. Discussion

**Figure 9:**
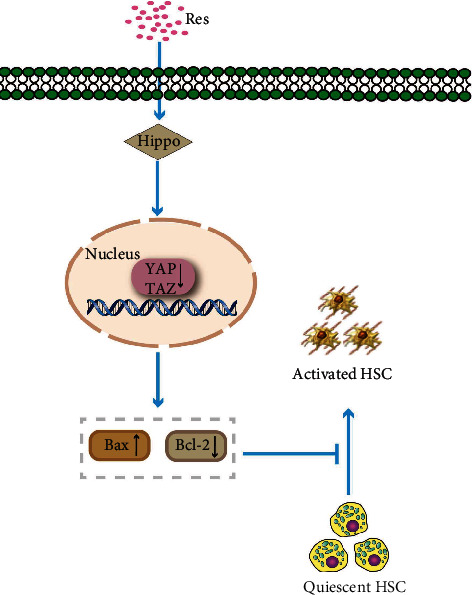
Schematic representation of the working model by which Res suppresses. HSC activation via the Hippo pathway.

The results of the current study show that Res inhibits HSC activation, at least partially, by activating the Hippo pathway. Res activates the Hippo pathway, thereby enhancing HSC apoptosis, which results in HSC inactivation (Figure 9). Our results reveal the involvement of Hippo-mediated HSC apoptosis in suppressing HSC activation and demonstrate a novel antifibrotic mechanism of Res. To our knowledge, our study is the first to report this.

Liver fibrosis is a compensatory response to a variety of chronic liver injuries. It results from an imbalance between ECM synthesis and degradation, and it is a common pathological process in end-stage liver diseases. Res, the predominant active component of *Polygonum cuspidatum*, has been demonstrated to provide beneficial effects in various human diseases such as cancers and fibrotic diseases [26]. For example, Res suppresses the survival and proliferation of gastric cancer cells via the inactivation of PIM-1 kinase activity [27]. Zou et al. demonstrated that Res can attenuate cardiac fibrosis via the PTEN/AKT/Smad2/3 and NF-*κ*B pathways [28]. In addition, Res has been reported to suppress liver fibrosis via the NF-*κ*B pathway as well as the PI3K/Akt pathway [9]. Recently, Li et al. found that Res alleviates liver fibrosis by inducing cell apoptosis [29]. In line with these findings, the present study showed that Res inhibits HSC activation by inducing cell apoptosis. Res treatment led to the downregulation of Bcl-2 and upregulation of Bax via the Hippo pathway, finally resulting in HSC apoptosis. Notably, we found that the inhibition of the Hippo pathway blocked the effect of Res on HSC apoptosis. In addition, Res-induced HSC inactivation was attenuated by Hippo pathway inhibition. Taken together, our data suggest that the Hippo pathway mediates the antifibrotic effects of Res.

The Hippo pathway restricts YAP/TAZ activation by retaining them in the cytoplasm through the activation of a phosphorylation cascade [30]. Once the Hippo pathway is activated, YAP and TAZ are phosphorylated by LATS1/2 and then retained in the cytosol, where they are degraded via the ubiquitin–proteosome pathway [31]. Abnormalities in the Hippo pathway can result in YAP/TAZ hyperactivation, which may contribute to the occurrence and development of many human diseases, including inflammation, fibrosis, and cancer [14, 32]. For instance, abnormal Hippo pathway activation has been observed during breast cancer tumorigenesis [33]. It has recently been reported that the Hippo/Yap pathway plays a key role in hepatic fibrogenesis [16]. In our study, KEGG pathway analysis indicated that the Hippo pathway may be involved in the effects of Res on HSC inactivation. Subsequently, the expression levels and roles of Hippo pathway-related genes after Res treatment were explored. We found that YAP and TAZ levels were reduced by Res treatment, suggesting that the Hippo pathway was activated in Res-treated cells. The decrease in the YAP and TAZ expression was also confirmed using immunofluorescence analysis. Interestingly, the level of YAP, reduced after Res treatment, could be restored by the Hippo pathway inhibitor XMU-MP-1. Further experiments showed that the inhibition of the Hippo pathway blocked the Res-mediated enhancement of HSC apoptosis, leading to the restoration of HSC activation. Accordingly, similar effects were noted in Res-treated cells overexpressing YAP. Our results demonstrate that Res suppresses HSC activation, at least in part, through Hippo-mediated HSC apoptosis. However, the mechanisms underlying the direct regulation of YAP/TAZ by Res remain to be explored. We hypothesize that microRNA-mediated effects on the Hippo pathway may be involved in the effects of Res treatment on liver fibrosis, and further studies are warranted.

## 5. Conclusion

Our results suggest that Res inactivates HSCs, at least partially, via Hippo-mediated HSC apoptosis. These results also indicate a new antifibrotic mechanism of Res and provide novel insights into Hippo-mediated HSC apoptosis and HSC activation in liver fibrosis.

## Data Availability

All datasets generated for this study are included in the manuscript files.

## Abbreviations

*α*-SMA: *α*-Smooth muscle actin
CCl_4_: Carbon tetrachloride
Col1A1: Alpha-1(I) collagen
Cur: Curcumin
DAPI: 4,6-Diamidino-2-phenylindole
DEGs: Differentially expressed genes
ECM: Extracellular matrix
HSC: Hepatic stellate cell
Res: Resveratrol
RNA-seq: RNA-sequencing
YAP: Yes-associated protein
TAZ: Transcriptional coactivator with PDZ-binding motif
qRT-PCR: Quantitative real-time PCR
H&E: Hematoxylin and eosin.
